# A Taxonomically-verified and Vouchered Checklist of the Vascular Plants of the Republic of Guinea

**DOI:** 10.1038/s41597-023-02236-6

**Published:** 2023-05-26

**Authors:** George Gosline, Ehoarn Bidault, Xander van der Burgt, Daniel Cahen, Gill Challen, Nagnouma Condé, Charlotte Couch, Thomas L. P. Couvreur, Léo-Paul M. J. Dagallier, Iain Darbyshire, Sally Dawson, Tokpa Seny Doré, David Goyder, Aurélie Grall, Pépé Haba, Pierre Haba, David Harris, D. J. Nicholas Hind, Carel Jongkind, Gbamon Konomou, Isabel Larridon, Gwilym Lewis, Alexandra Ley, Michael Lock, Eve Lucas, Sékou Magassouba, Simon Mayo, Denise Molmou, Alexandre Monro, Jean Michel Onana, Jorge Paiva, Alan Paton, Sylvia Phillips, Ghillean Prance, Alejandro Quintanar, Saba Rokni, Toral Shah, Brian Schrire, André Schuiteman, Ana Rita Giraldes Simões, Marc Sosef, Tariq Stévart, R. Doug Stone, Tim Utteridge, Paul Wilkin, Martin Xanthos, Eimear Nic Lughadha, Martin Cheek

**Affiliations:** 1 Royal Botanic Gardens, Kew, Richmond, UK; 2 Missouri Botanical Garden, St. Louis, USA; 3 Herbier National de Guinée, UGAN-Conakry, Conakry, Guinea; 4 DIADE, Univ Montpellier, CIRAD, IRD, Montpellier, France; 5 Naturalis Biodiversity Centre, Botany Section, Leiden, The Netherlands; 6 New York Botanical Garden, Bronx, USA; 7 University of Basel, Basel, Switzerland; 8 Royal Botanic Garden, Edinburgh, Scotland; 9 Meise Botanic Garden, Meise, Belgium; 10 Martin Luther University, Halle, Germany; 11 Université de Yaoundé 1, Cameroon; IRAD-Herbier National Camerounais, Yaoundé, Cameroon; 12 University of Coimbra, Coimbra, Portugal; 13 Real Jardín Botánico de Madrid CSIC, Madrid, Spain; 14 University of KwaZulu-Natal, Durban, South Africa

**Keywords:** Biodiversity, Plant sciences

## Abstract

The Checklist of the Vascular Plants of the Republic of Guinea (CVPRG) is a specimen-based, expert-validated knowledge product, which provides a concise synthesis and overview of current knowledge on 3901 vascular plant species documented from Guinea (Conakry), West Africa, including their accepted names and synonyms, as well as their distribution and status within Guinea (indigenous or introduced, endemic or not). The CVPRG is generated automatically from the Guinea Collections Database and the Guinea Names Backbone Database, both developed and maintained at the Royal Botanic Gardens, Kew, in collaboration with the staff of the National Herbarium of Guinea. A total of 3505 indigenous vascular plant species are reported of which 3328 are flowering plants (angiosperms); this represents a 26% increase in known indigenous angiosperms since the last floristic overview. Intended as a reference for scientists documenting the diversity and distribution of the Guinea flora, the CVPRG will also inform those seeking to safeguard the rich plant diversity of Guinea and the societal, ecological and economic benefits accruing from these biological resources.

## Background & Summary

The conservation of biological diversity is a common concern of humankind, but states have sovereign rights over their own biological resources and are responsible for conserving their biodiversity and using their biological resources sustainably (Convention on Biological Diversity, https://www.cbd.int). A consequence of these rights and responsibilities is that each state needs an overview of the species present within their borders. Development and refinement of such inventories for plants was a major focus in the early 21st century, in response to the CBD’s Global Strategy for Plant Conservation^1,2^.

Many tropical countries face great challenges in preparing national plant inventories. The tropics are home to most of the c. 350,000 known vascular plant species, but their plant diversity is relatively poorly-documented, and vital reference materials are often inaccessible in herbaria overseas. Thus, herbarium research or fieldwork regularly yields additions to the known flora: either species new to science^3–8^ or species known to science but documented for the first time in the country of interest.

The Checklist of Vascular Plants of the Republic of Guinea (CVPRG) is a comprehensive listing of all 3505 vascular plant species documented as occurring naturally within Guinea (3328 flowering plants, 177 pteridophytes). Also listed are 396 non-indigenous species recorded in Guinea, but introduced plants are less frequently collected so coverage is incomplete. For each vascular plant species reported from Guinea, we aimed to cite at least one expert-verified, collection-based record, including all taxa recorded in the Flore de la Guinée^9^ which documented 2633 indigenous angiosperm species. These voucher specimens provide auditable evidence of the occurrence of each taxon in Guinea.

The CVPRG is a specimen-based, expert-validated knowledge product, generated automatically from the Guinea Collections Database and the Guinea Names Backbone Database, both developed and maintained at the Royal Botanic Gardens, Kew (hereafter Kew), in collaboration with the National Herbarium of Guinea. Earlier iterations of CVPRG underwent expert review by 42 regional or taxon specialists who co-authored this data descriptor. The CVPRG is published in two formats: a Darwin Core Archive available from GBIF^10^ and a printable pdf available from Zenodo^11^.

The Guinea Collections Database is based on an extract from Kew’s Wet Tropics Africa database comprising records from environmental surveys for mining projects 2005–2015, records from botanical exploration and Red Listing activity in relation to Tropical Important Plant Areas^12^ and specimen records cited by^9^ of taxa not represented in other sources. These records were complemented by: (i) data from GBIF for specimens deposited in herbaria at Kew, Missouri Botanical Garden and Muséum National d’Histoire Naturelle, Paris (K, MO, P; codes follow Index Herbariorum (Thiers, B.M. Index Herbariorum. http://sweetgum.nybg.org/science/ih/)); (ii) data from P georeferenced via the Guinea Biodiversity Information for Development (BID) project^13–15^; data from MO, and from the herbaria of Meise Botanic Garden (BR) and Naturalis Biodiversity Centre (WAG) informed by the Rainbio database^16^.

The Guinea Names Backbone Database is based on a download from the African Plant Database (APD (version 4.0.0) Conservatoire et Jardin botaniques de la Ville de Genève and South African National Biodiversity Institute, Pretoria, http://africanplantdatabase.ch (accessed 29 July 2021)) for tropical Africa. This was complemented by records from Plants of the World Online (POWO. Facilitated by the Royal Botanic Gardens, Kew. http://www.plantsoftheworldonline.org/2019-2022) for all taxa reported in^9^.

Guinea is unusual among tropical African countries in having a recently published Flora^9^, but all data therein are over 30 years old, mostly over 50 years old. The first decades of the 21^st^ century saw rapid growth in botanical fieldwork in Guinea, yielding numerous herbarium specimens documenting the Guinean flora. However, this period saw major tree-cover loss (25% in 2000–2018) in the Forestière region, home to Guinea’s greatest known plant diversity^17^. Against a backdrop of habitat loss due to subsistence and cash-crop farming, exacerbated by growing threats from mining, CVPRG is a key resource for those researching, communicating about and seeking to safeguard Guinea’s rich plant diversity and the social, ecological and economic benefits accruing from these biological resources. CVPRG provides an evidence-base for prioritising species for extinction risk assessment, and for species- or area-focused action plans. It will inform Guinea’s forthcoming biodiversity management plan and serve both as a foundation for future research and a baseline against which change in Guinea’s flora can be monitored.

## Methods

### Geographic and taxonomic scope

#### Study area

**Fig. 1:**
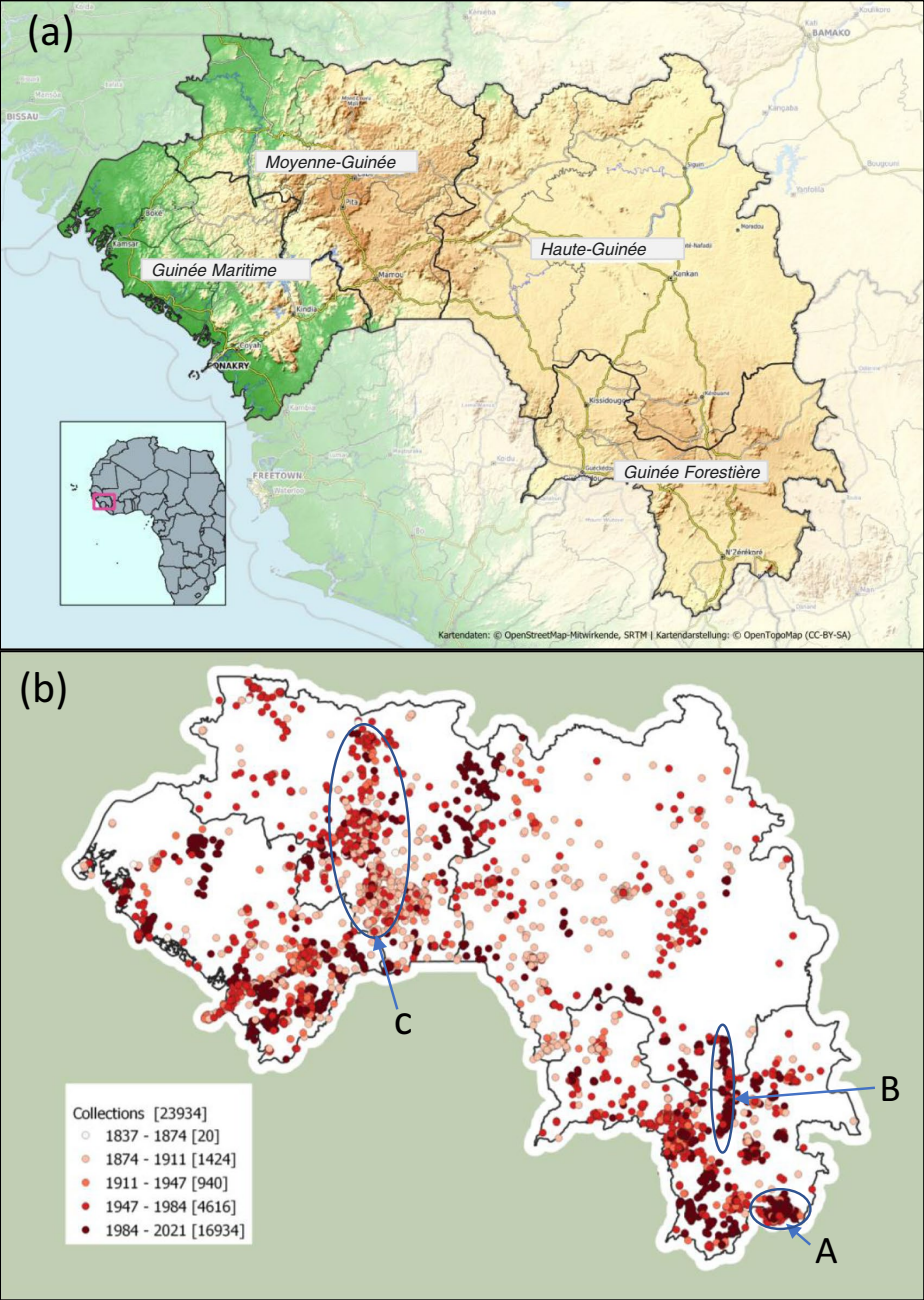
Maps showing Guinea’s location, political and geographic features (**a**), and distribution of botanical collections in space and time (**b**). (**a**) Map showing location in West Africa of Republic of Guinea and the four regions in which it is divided. Inset shows Guinea’s location in West Africa. Main map shows boundaries of Guinea’s four regions which are used to summarise species distributions in the Checklist of Vascular Plants of the Republic of Guinea. (b): Map of collection locations of the georeferenced collections in the Guinea Collections Database. Spatial biases in collecting patterns include the following collecting ‘hotspots’: **A** Mts Nimba; **B** Mts Simandou; **C** around the major towns of the Fouta Djallon: Mamou, Dalaba, Pita, Labe and Mali.

The geographic scope of our study is the Republic of Guinea (245,857 km^2^), also known as Guinea-Conakry, formerly Guinée Française or French Guinea, situated in West Africa (Fig. 1). The country is dominated by the Guinea Highlands. They form or influence the vegetation types that give Guinea its diverse flora and have many narrow endemics^18^. The Highlands are the highest and most extensive in coastal continental Africa west of the Cameroon Highlands which lie 2000 km to the east. The Guinea Highlands rise to between 1000–2000 m above sea-level and are divided into two portions, separated by a 500 m elevation saddle that coincides with the western frontier of Sierra Leone with Guinea. Major rivers such as the Niger, Senegal and Mano arise in the Highlands, hence the country is known as the “water tower” of West Africa. Rainfall is monsoonal, falling mainly in May-October. The highest rainfall occurs at the coast, Conakry (4000 mm/a) falling to 1000 mm/a near the border with Mali in the north. In the northwest, the Fouta Djallon highlands are entirely confined to Guinea except for some foothills in Mali and Senegal. The Fouta Djallon are sandstone table mountains with some lateritic bowal (grassland areas lacking trees). This formation was formerly connected with the American Guiana Shield and its tepuis before the opening of the Atlantic. The geologically different Loma-Man Highlands to the southeast fall mainly into Guinea, but extend from Mts Loma in northern Sierra Leone to the Man Mts of western Ivory Coast. The well-known Nimba mountains straddle the borders between Ivory Coast, Guinea, and Liberia.

The dominant intact vegetation types of Guinea are woodland and grassland. Much of the latter occurs on lateritic or sandstone hardpans (bowal) which are widespread and common, and sometimes seasonally inundated. Granitic inselbergs occur in clusters scattered across the country. The native vegetation is very reduced and fragmented, and agriculture is extensive. 96% of original forest was recorded as lost before the end of the 20^th^ century^19^ and Guinea burns from end to end in the dry season due to fires set to promote forage for livestock. Wetland areas have been extended and modified for cultivation of West African rice (*Oryza glaberrima* Steud.), the traditional staple crop.

#### Taxonomic scope and biogeographic interest

Our study aims to encompass the vascular plants known to date from Guinea, highlighting the unique and relatively little-studied flora of this country which is perhaps best known to botanists for its amphi-Atlantic plant taxa. Most famously, the only indigenous Bromeliaceae species not restricted to the New World, *Pitcairnia feliciana* (A. Chev.) Harms & Mildbr. uniquely occurs in Guinea, as a localised sandstone cliff endemic of the Fouta Djallon. Less well-known is that the lowland wetland species *Maschalocephalus dinklagei* Gilg & K. Schum., the only Rapateaceae species not restricted to the New World, also occurs in Guinea, as well as in neighbouring Sierra Leone and Liberia^20^. Similarly, molecular phylogenetic data have shown that *Fleurydora felicis* A. Chev. a monotypic tree also endemic to sandstone cliffs, is the only African representative of an otherwise New World clade of Ochnaceae^21^. It is likely that further molecular phylogenetic research will uncover more such amphi-Atlantic disjunctions such as the recent discovery that *Soyauxia* Oliv. (formerly Medusandraceae) is in fact an African member of the Peridiscaceae, until then considered endemic to S America^22,23^.

### Baseline resources and rationale for methodological choices

#### Existing documentation of Guinea vascular plants

The Flora of Guinea was considered well-known due to the surveys that began in 1837 with the collections of Heudelot (which were often misleadingly labelled as Senegambia). During the French colonial period (1898–1958) sampling was expanded by collectors such as Chevalier, Jacques-Félix, Adam and Schnell who also published new records and taxa new to science based on their collections and those of others. The culmination of this work was the Flore (Angiospermes) de la République de Guinée^9^ which is largely extracted from the Flora of West Tropical Africa^24^, greatly augmented by c. 4000 additional records made by Lisowski in the post-colonial period (1958 onwards) when Guinea was supported by the Soviet Union. Lisowski completed his Flora in 2000 but publication was delayed until 2009. He mainly followed the generic and family concepts of^24^, and recorded 2633 indigenous species of angiosperms and a further 395 cultivated or introduced angiosperm species.

**Fig. 2:**
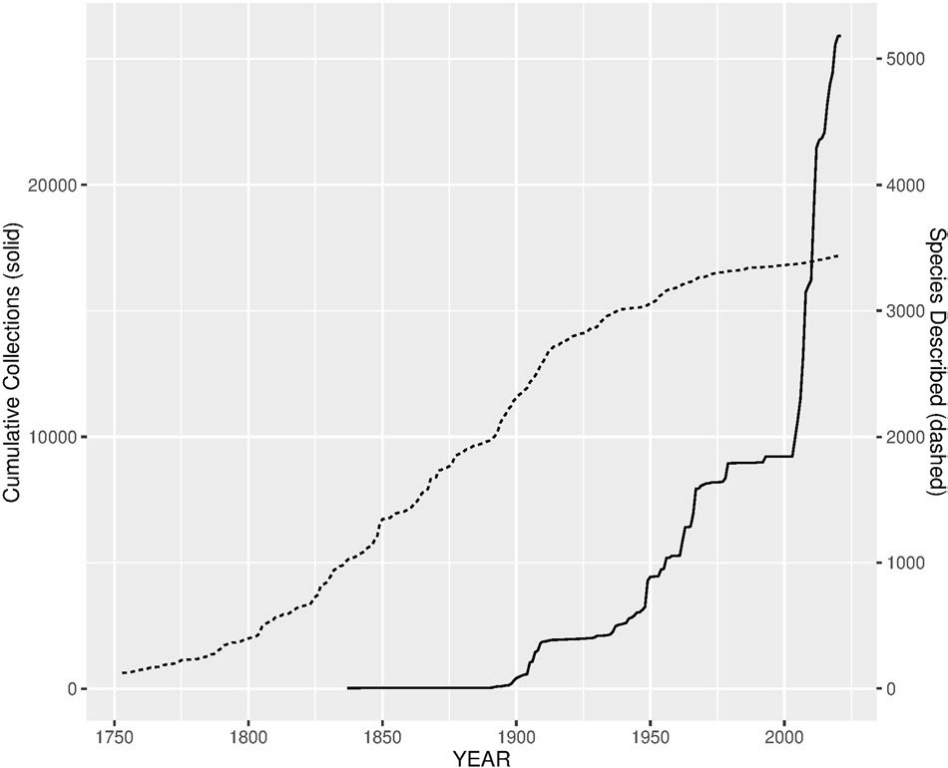
Growth in numbers of plant species known from Guinea and in the numbers of herbarium specimens documenting the Guinea flora. Increase over time in: (i) plant species recorded from the Republic of Guinea, plotted (dashed trace) by the date when species was first described (which may predate their first record from Guinea by many years) and (ii) herbarium specimens collected in Guinea and incorporated in the Guinea Plant Collections database, plotted (solid trace) by their year of collection.

However, botanical surveys in the 21^st^ century, principally in the Loma-Man of Guinea in connection with proposed mining projects in or near the Simandou and Nimba ranges, increased the numbers of herbarium specimens and of recorded plant species known for Guinea^25–29^ (Fig. 2). This continued with the initiation in 2016 of the Guinea Tropical Important Plant Areas (TIPA) programme^30–33^, which seeks to identify, document and delineate areas of particular importance for plant conservation within Guinea.

#### Choice of specimen-based approach

It is possible to extract plant species checklists for countries or regions of interest from global resources such as Plants of the World Online (http://www.plantsoftheworldonline.org) but such lists can inflate species numbers^34^, and are not usually verifiable by means of voucher specimens cited for each area from which the species is reported (specimen-based). Therefore, development of an updated, expert-verified, specimen-based checklist with authoritatively updated nomenclature was the preferred approach to provide a solid, evidence-based foundation both for future research and for biodiversity management. We sought to report all vascular plant species known to occur naturally within Guinea, each supported by at least one authoritatively identified voucher specimen collected within Guinea, deposited in a collection and accessible to researchers, physically and/or digitally.

### Data acquisition and processing

Creating a comprehensive, contemporary specimen-based checklist of a region requires two parallel workflows:

- Gathering all available data on field collections within the region and validation of identifications.
- Creating a “names backbone” of accepted taxon names and synonyms.

Here we describe data acquisition and processing for each of these workflows, before reporting how they were brought together for iterative checklist creation and expert validation steps.

#### Collections data acquisition

Plant specimen data was collected from a variety of existing digital resources including herbarium collection databases at the Royal Botanic Gardens, Kew (K), the Missouri Botanical Garden (MO), and the MNHN, Paris (P), the herbarium at Adam Mickiewicz University (POZG), the herbarium of the Naturalis Biodiversity Centre (WAG), from GBIF^35–38^, and from datasets developed for earlier projects including Guinea BID^13–15^, Guinea TIPAs and Rainbio^14^. Data were also extracted from Lisowski’s Flore de la Guinée^9^. All our collections data sources are detailed in Table 1. This data was reduced to a common record format and consolidated in a Microsoft Access database, the Guinea Collection Database comprising 32330 records.

**Table 1:** Summary of sources of collections data recorded in the Guinea Collections Database, from which the Checklist of Vascular Plants of the Republic Guinea was compiled.

Sources for specimens recorded in Guinea Collections database	Number of Records
P	6067
K	11919
WAG	3605
POZG	3502
MO	2998
BR	187
RainBio^16^ record - specimen not seen	2365
Lisowski Flora^9^ - no herbarium catalogue number	1034
Taxonomic specialists (including Roux^62^)	35

Herbarium codes follow Index Herbariorum (Thiers, B.M. Index Herbariorum. http://sweetgum.nybg.org/science/ih/)): P = Muséum National d’Histoire Naturelle, Paris; K = Royal Botanic Gardens, Kew; WAG = Naturalis Biodiversity Centre, Leiden; POZG = Adam Mickiewicz University, Poznan; MO = Missouri Botanical Garden; BR = Meise Botanic Garden.

We aimed to ensure that collections coverage in this database was sufficient to have at least one expert-verified collection-based record cited of each vascular plant taxon found in Guinea, including all taxa recorded in the Flore de la Guinée^9^. A secondary objective was to obtain an idea of the geographic range of each taxon within Guinea (28164 collections are georeferenced at varying levels of accuracy). We have not attempted to record in our database every plant collection ever made in Guinea nor to georeference all specimens, an endeavour beyond the scope of the project.

#### Collections data processing

All taxon names obtained from specimens are standardised to accepted names based on our names backbone database (see below). When specimens are collected, duplicates are generally sent to several herbaria. There may be several specimen records for any given collection event and therefore individual plant. A process of deduplication based on matching collection number, taxon name, and date was performed; a preferred duplicate was selected with the most complete metadata. 1486 duplicate records have been excluded from the analysis.

#### Names data acquisition

A custom names backbone database was built, the Guinea Names Backbone Database. The starting point was a download in 2019 from the African Plant Database (APD (version 4.0.0), Conservatoire et Jardin botaniques de la Ville de Genève and South African National Biodiversity Institute, Pretoria, http://africanplantdatabase.ch (accessed 29 July 2021)) for the tropical Africa area. From Plants of the World Online (POWO, facilitated by the Royal Botanic Gardens, Kew. http://www.plantsoftheworldonline.org/2019-2022) records for all taxa in^9^ and all taxa shown as present in Guinea were downloaded and the information matched by taxon name and added to the Guinea Names Backbone Database. Data records have been added for taxa new to science as they have been published. Resources consulted during compilation included publications describing species or genera new to science^39–42^; other taxonomic literature such as taxonomic monographs, revisions, synopses or checklists^43–66^; phylogenetic studies^67–75^; nomenclatural updates^76–81^; collectors’ biographies or itineraries^82,83^; and the following online data resources resulting from earlier and/or ongoing compilation endeavours: the BioPortal of Naturalis Biodiversity Centre (https://bioportal.naturalis.nl): the Botanical Collections resource hosted by Meise Botanic Garden (http://www.botanicalcollections.be/#/en/home); the AMUNATCOLL Database of Natural History Collections of the Faculty of Biology Adam Mickiewicz University, Poznan (https://amunatcoll.pl); Hassler, M. (2004 - 2021): World Ferns. Synonymic Checklist and Distribution of Ferns and Lycophytes of the World. Version 12.4 (https://www.worldplants.de/world-ferns/ferns-and-lycophytes-list); Missouri Botanical Garden’s Tropicos (http://www.tropicos.org/Home.aspx).

#### Names data processing

Lisowski’s Flore (Angiospermes) de la République de Guinée^9^ was taken as a starting point for synonymy in the printable version of the CVPRG^11^. We endeavoured to ensure that any taxon name that is accepted by Lisowski^9^ or is a current specimen identification, but which is no longer considered an accepted name, is included in synonymy in CVPRG. This pragmatic decision was based on the primary objective: to produce a useful checklist reflecting current taxonomy, and it follows established practice in the region^84–90^. More comprehensive synonymy data based on 31,357 synonyms derived from World Checklist of Vascular Plants (now available via POWO https://powo.science.kew.org/about-wcvp) was incorporated in the Darwin Core Archive version of the CVPRG downloadable from GBIF^10^.

### Checklist generation and expert validation

#### Names data expert validation

The expert opinions of 42 checklist authors, each specialising in a different taxonomic group or in the flora of the region as a whole (Supplementary Table 1), were used to clean the Guinea Names Backbone Database. Where sources disagreed as to the accepted name and synonymy, a choice has been made by the specialist concerned. For groups where no specialist was available (e.g. Moraceae, Vitaceae), APD was followed.

#### Collections data expert validation

Collection records from the Guinea Collections Database were sent to the expert reviewers along with draft checklist family reports. Experts were not asked to confirm every collection determination, but rather to review taxa with few collections (generally three or fewer) or outside of recorded distributions shown on POWO. The vast majority of taxon name changes since 2010 are due to nomenclatural changes. Guinea species which lacked taxonomist-verified records from any other country were assigned as endemics (Supplementary Table 2) after checking herbarium records, APD, POWO, GBIF, and taxonomic literature^9,20,26,30,31,91–104^.

#### Generation of the checklist

The checklist is generated automatically from the Guinea Collections Database and the Guinea Names Backbone Database. This prevents the databases and text copy from getting out of sync. The collections are grouped and summarized using Access queries (SQL) and exported to an XML file. This file was then used to generate versions of the checklist in Word document form for publication as a printable pdf^11^ and as Darwin Core data^10^ which was uploaded to GBIF using their Integrated Publishing Toolkit (IPT, https://www.gbif.org/ipt), applying GBIF guidelines for checklist publication (https://ipt.gbif.org/manual/en/ipt/latest/checklist-data).

Each expert screened and annotated draft checklist accounts of the taxa in which they specialise (Supplementary Table 1) in late 2019, in mid-2021 and finally, in mid-2022. Final updates to the checklist were made in August 2022. All changes were incorporated in the Guinea Collections Database and Guinea Names Backbone Database before the creation of the printable version of the checklist^11^ and the corresponding Darwin Core dataset^10^.

#### Structure of the printable checklist

Data records from the Guinea Collections Database and Guinea Names Backbone Database are grouped and summarised to create family lists comprising one or more species treatments. Within the checklist, the angiosperm families are ordered following Angiosperm Phylogeny Group IV^105^, while the Pteridophytes follow WorldFerns (Hassler, Michael (2004 - 2022): World Ferns. Synonymic Checklist and Distribution of Ferns and Lycophytes of the World. Version 14.2 -www.worldplants.de/ferns/).

Each species treatment includes the accepted species name and, where applicable, selected synonyms. Species treatments also include: an indication as to whether the species is considered endemic to Guinea or introduced to Guinea; voucher information in the form of key details for one preserved specimen, representing auditable evidence of the presence of the species in Guinea; followed by geographic distribution data summarising the frequency of collection records from each of the major regions of Guinea.

Within each family, accepted names of indigenous and introduced species are ordered alphabetically in a single sequence by genus and specific epithet. Selected synonyms, if any, appear immediately below their accepted name. Data for infraspecific taxa (subspecies or varieties) appear below the corresponding species entry. Details of publishing authors follow each name at species or infraspecific rank, except for autonyms.

The most recent specimen in the Guinea Collections Database of each species or infraspecific taxon is generally presented as a voucher. The voucher information usually comprises the collector(s’) name(s) and collecting number, followed by the date of collection and, where available, an ID unique to that specimen (usually a barcode). This allows users to rapidly find and view any corresponding specimen details and images made available by the herbarium where the specimen is deposited (indicated by an alphabetical prefix to the ID number) or via an aggregator such as GBIF. No digital record of a herbarium specimen has been located for 642 species. 157 species are vouchered by K specimens which have not yet received a barcode (indicated by “WTA” in the barcode field). 413 taxa are vouchered by specimens cited in Lisowski^9^, indicated by “Lisowski” or “Berhaut” in the barcode field. The majority of these have collection information, with the specimens believed to be at Paris (P) where, although digitised, specimen images are not yet available for searching by country, due to minimal transcription of label data. Future work is planned to locate and validate the Paris specimens. 28 fern taxa are included based on distribution data in Roux’s Synopsis of the Lycopodiophyta and Pteridophyta of Africa, Madagascar and neighbouring islands^62^ and therefore marked as “lit.”. A few collections are supplied by researchers with no barcodes. Where no specimen is known to exist, an observation reported by Lisowski^9^ is listed in place of a voucher citation. 75 species are included based solely on observations recorded in Lisowski^9^. Of these 63 are introduced species. Thus, all but 12 indigenous angiosperm species are vouchered.

Each species treatment ends with a summary of the relevant specimens available for Guinea as a whole (‘Total’), followed by a breakdown of collections number for each of the four geographic regions of Guinea: Guinée Forestière (GF), Haut Guinée (HG), Moyenne Guinée (MG), Guinée Maritime (GM, = Basse Guinée (GN-BA) in^10^) (See Fig. 1a). These collection counts give an indication of the relative frequency of each species across Guinea’s regions but should be considered with caution in light of known biases in sampling intensity (Fig. 1b). Collection counts for species and their infraspecific taxa may differ where some collections are identified to species-level but not to variety or subspecies.

### Statistical overview of the checklist

The CVPRG records a total of 3505 indigenous species known from Guinea, of which 3328 are angiosperms and the remaining 177 are pteridophytes. Thus, the total number of angiosperms considered indigenous to Guinea is now 26% greater than the 2633 species reported by Lisowski^9^. In total, 81 species and one subspecies are reported as endemic to Guinea (i.e. 2% of the known flora). A further 26 species and one subspecies previously considered endemic to Guinea are reported as occurring in other countries (See Supplementary Table 2 for a complete list of endemic species, and those previously considered endemic, with life form and habitat for each). Of the 39 species and one subspecies occurring in Guinea which have been reported as new to science since Lisowski’s Flora^9^, 14 (35%) are known only from Guinea and six of these have been assessed as Endangered or Critically Endangered on the IUCN Red List of Threatened Species. The remaining eight species await assessment. (See Supplementary Table 3 for a complete list of these species with habitat, IUCN category (where available) and distribution beyond Guinea (where applicable)).

## Data Records

The download of the data set(s) is available via the GBIF.org repository under a CC-BY 4.0 Licence in a Darwin Core Archive File.

The CVPRG has been published as a Checklist dataset^10^ on GBIF. The data may be examined and downloaded from GBIF: 10.15468/f5gb45.

Darwin Core Archive (DwC-A) is a standardized format for sharing biodiversity data as a set of one or more data tables. The Taxon core data table contains 34,048 records, each with the data fields listed in Table 2. These comprise 4,195 accepted names and 29,821 synonyms. The IPNI plant ID (https://www.ipni.org) is used as the stable identifier for taxa.

**Table 2:** Content of Taxon core data download available from GBIF.

DwC Taxon core	Notes
Field Name	
id	Taxon identifier. The numeric portion of the IPNI Life Sciences Identifier (LSID) for the taxon.
taxonID	The taxon identifier. Same as the id field above.
family	The name of the family to which the taxon belongs.
specificEpithet	The species epithet which is combined with the genus name to make a binomial name for a species
infraSpecificEpithet	The infraspecific epithet which is combined with a binomial to make a trinomial name at infraspecific rank, most commonly a subspecies or variety
scientificName	Concatenation of genus with species and, where applicable, infraspecific epithets to make a binomial or trinomial name
scientificNameAuthorship	The author or authors responsible for publication of the scientific name
taxonRank	The level in the taxonomic hierarchy where the taxon name fits
taxonomicStatus	Indication of taxonomic opinion re the name: accepted, synonym or unplaced
acceptedNameUsageID	Taxon number (taxonID) of the accepted name for a synonym (found in this same file).
parentNameUsageID	taxonID of the species record for varieties and subspecies.

Field names (which appear as column headers in the download) and explanatory notes on their content.

Two extension data tables are also part of the DwC Archive. An extension record supplies extra information about a core record.

The Occurrence extension contains one record per taxon, that of the voucher specimen chosen to evidence the presence of the taxon within Guinea. It comprises 4024 voucher records in total, each with the data fields listed in Table 3.

**Table 3:** Content of Taxon Occurrence data download available from GBIF.

DwC Taxon Occurrence extension	Notes
Field Name	
id	taxonID of associated taxon
basisOfRecord	Always “MaterialCitation” indicating a voucher
occurrenceID	Unique identifer for occurrence record
catalogNumber	Barcode of the specimen when available. First letters indicate herbarium where specimen is deposited.
	For specimens lacking barcodes, “Lisowski” indicates source is Lisowski’s Flora.
recordNumber	Collector’s collection number
recordedBy	Collector
eventDate	Date of collection
countryCode	Always GN
decimalLatitude	When available
decimalLongitude	When available
taxonID	taxonID of the associated taxon. (Same as id)
scientificName	Full scientific name without author

Field names (which appear as field headers in the download) and explanatory notes on their content.

The Species Distribution extension contains one or more records per taxon: one for the country and one for each region within Guinea from which the Guinea Collections database contains at least one specimen of the taxon, making 10419 records in total, each with the data fields listed in Table 4.

**Table 4:** Content of Taxon Species Distribution data download available from GBIF.

DwC Taxon Species Distribution extension	Notes
Field Name	
id	taxonID to which the record refers
locationID	Region of Guinea from which specimen of taxon is in Guinea Collections Database:
	GN-BA Basse-Guinée
	GN-FO Guinée Forestière
	GN-HA Haute-Guinée
	GN-MO Moyenne-Guinée
locality	Basse-Guinée
	Guinée Forestière
	Haute-Guinée
	Moyenne-Guinée
countryCode	Always GN
establishmentMeans	“native” or “introduced” Note that many introduced species are now naturalized.

Field names (which appear as field headers in the download) and explanatory notes on their content.

Together, the data in the three data tables described above (Tables 2–4) include all the data presented in the CVPRG with the following exceptions: (i) the indication of species considered endemic to Guinea; (ii) the numbers of specimens of each species known from Guinea as a whole and from each of the four regions of Guinea. These omissions are due to the limitations of the Darwin Core format.

## Technical Validation

The checklist was built and formatted directly from the Guinea Collections Database, avoiding transcription errors. Where three or fewer collections exist for a taxon, the specimens have been examined by taxonomic experts to validate the identifications.

Our Guinea Names Backbone Database was constructed based on a download of the African Plant Database 15/01/2019. Data from POWO for taxa in the TDWG Guinea area were downloaded and matched to the APD data. Where the two sources did not match, the experts’ opinions were used to select an accepted name. Further names from recent literature have been incorporated (See under Names Data Acquisition above). In all cases, the opinions of the checklist authors have been followed.

All names were automatically checked against our Guinea Names Backbone Database. This process resolved all names to the accepted name for the checklist. Any synonyms found to be current on specimens were automatically included in the synonyms list.

Authors and basionyms were copied from the Guinea Names Backbone Database, again avoiding transcription errors. Where homonyms exist (identical binomials or trinomials intended to refer to different entities), the collection and geographic range were checked to verify the correct author string.

Collections were automatically assigned to geographic region based on their geographic coordinates available. All collections bearing geolocation data beyond Guinea’s borders were checked and either their coordinates were corrected, or the collections were excluded from the checklist.

## Usage Notes

Like almost any resource documenting a tropical flora, the CVPRG will be out of date almost as soon as it is published. In fact, publication of a checklist has been shown to stimulate publication of further research on the flora documented. In Guinea, new data on the flora can be expected to include: descriptions of species new to science; records of species already known to science but recorded for the first time in Guinea; new perspectives on the endemism of species currently known only from Guinea; assessments of the extinction risk of species occurring in Guinea; reports on cultural and economic uses of Guinea plants.

Depending on their reasons for consulting the CVPRG, potential users may also wish to consult additional resources to obtain as complete a picture as possible on one or more of the above aspects for the subset of species of interest to them. Here we itemize some of the key resources of relevance for obtaining updates on the different facets of the data.

### Species described as new to science

Description of species new to science^3–8,20,25–29,31–33,39,48,49,55,62,66,92,97–100,103,104,106–112^ has accelerated in Guinea in the past 10 to 15 years (see Supplementary Table 3 for examples) and this trend looks set to continue. Users wishing to find details of Guinea species described as new to science post-dating our work should consult the International Plant Names Index (IPNI. International Plant Names Index. http://www.ipni.org, The Royal Botanic Gardens, Kew, Harvard University Herbaria & Libraries and Australian National Botanic Gardens 2022) in the first instance, as newly published species are indexed there throughout the year and the web resource is updated daily. These newly described species will also appear in APD and POWO eventually.

### Species newly recorded as present in Guinea

The majority of the growth in numbers of indigenous species known from Guinea arises from extensions to the known range of species already known from elsewhere in Africa. The specimens evidencing such changes to known ranges may be sought in aggregators such as GBIF or collection lists such as Tropicos. Such range extensions will be reflected in APD and POWO eventually.

### Corrections to the endemism status reported for Guinea plants

Corrections to the endemism status reported for Guinea plants may be sought in aggregators such as GBIF or collection catalogues such as Tropicos. A more fully informed view on the endemism status of many species must await completion of digitisation of the African collections at Kew and completion of digitisation of label data from MNHN (P).

### Assessments of the extinction risk of Guinea plants

Data on the extinction risk of many Guinea vascular plant species can be found on the IUCN Red List of Threatened Species (IUCN. 2022. The IUCN Red List of Threatened Species. Version 2022-1. https://www.iucnredlist.org), to which new assessments are added two or three times per year. Assessment efforts are ongoing, most recently within the framework of the Guinea Tropical Important Plant Areas programme.

### Overview and site-specific data

Data on any or all of the above topics may also be found in the data sheets which support recognition and delineation of Tropical Important Plant Areas in Guinea, available via the Tropical Important Plant Areas Explorer (Royal Botanic Gardens, Kew. Tropical Important Plant Areas Explorer tipas.kew.org)

## Supplementary information

Supplementary Table 1 Taxonomic specialists and the plant families they reviewed for CVPRG

Supplementary Table 2: Vascular plant species endemic to the Republic of Guinea

Supplementary Table 3: Recently described vascular plant species which occur in Guinea

## Data Availability

No custom code was used to generate this dataset.
